# Impaired ketogenesis is associated with metabolic-associated fatty liver disease in subjects with type 2 diabetes

**DOI:** 10.3389/fendo.2023.1124576

**Published:** 2023-02-22

**Authors:** Sejeong Lee, Jaehyun Bae, Doo Ri Jo, Minyoung Lee, Yong-ho Lee, Eun Seok Kang, Bong-Soo Cha, Byung-Wan Lee

**Affiliations:** ^1^ Division of Endocrinology and Metabolism, Department of Internal Medicine, CHA Gangnam Medical Center, CHA University School of Medicine, Seoul, Republic of Korea; ^2^ Division of Endocrinology and Metabolism, Department of Internal Medicine, Catholic Kwandong University College of Medicine, International St. Mary’s Hospital, Incheon, Republic of Korea; ^3^ Department of diabetes mellitus, Biomedical Research Center, Severance Hospital, Yonsei University College of Medicine, Seoul, Republic of Korea; ^4^ Division of Endocrinology and Metabolism, Department of Internal Medicine, Yonsei University College of Medicine, Seoul, Republic of Korea

**Keywords:** ketogenesis, β-hydroxybutyrate, steatosis, MAFLD, diabetes

## Abstract

**Aims:**

The ketogenic pathway is an effective mechanism by which the liver disposes of fatty acids (FAs) to the peripheral tissues. Impaired ketogenesis is presumed to be related to the pathogenesis of metabolic-associated fatty liver disease (MAFLD), but the results of previous studies have been controversial. Therefore, we investigated the association between ketogenic capacity and MAFLD in subjects with type 2 diabetes (T2D).

**Methods:**

A total of 435 subjects with newly diagnosed T2D was recruited for the study. They were classified into two groups based on median serum β-hydroxybutyrate (β-HB) level: intact *vs*. impaired ketogenesis groups. The associations of baseline serum β-HB and MAFLD indices of hepatic steatosis index, NAFLD liver fat score (NLFS), Framingham Steatosis index (FSI), Zhejian University index, and Chinese NAFLD score were investigated.

**Results:**

Compared to the impaired ketogenesis group, the intact ketogenesis group showed better insulin sensitivity, lower serum triglyceride level, and higher low-density lipoprotein-cholesterol and glycated hemoglobin levels. Serum levels of liver enzymes were not different between the two groups. Of the hepatic steatosis indices, NLFS (0.8 *vs*. 0.9, p=0.045) and FSI (39.4 *vs*. 47.0: p=0.041) were significantly lower in the intact ketogenesis group. Moreover, intact ketogenesis was significantly associated with lower risk of MAFLD as calculated by FSI after adjusting for potential confounders (adjusted odds ratio 0.48, 95% confidence interval 0.25-0.91, p=0.025).

**Conclusions:**

Our study suggests that intact ketogenesis might be associated with decreased risk of MAFLD in T2D.

## Introduction

Non-alcoholic fatty liver disease (NAFLD) is a liver condition ranging from simple steatosis to inflammation or fibrosis in the absence of excessive alcohol consumption (1). Hepatic triglycerides (TGs) accumulate in the liver mainly through *de novo* lipogenesis or delivery of fatty acids (FAs) from peripheral tissues in the NAFLD or insulin resistant settings (2, 3). Recently, metabolic-associated fatty liver disease (MAFLD) was suggested as a term that more accurately reflects the pathogenesis of this type of fatty liver disease (4).

In the normal liver, excess delivered FAs undergo β-oxidation, yielding acetyl-CoA (5). The majority of acetyl-CoA is condensed in the ketogenic pathway to form ketone bodies, mostly β-hydroxybutyrate (βHB) and acetoacetate, which are exported to the peripheral tissues and are utilized as efficient fuels. Through β-oxidation and the ketogenic pathway, the normal liver has considerable capacity to dispose of delivered FAs (6).

In this context, it can be expected that ketogenesis is relatively downregulated in patients with MAFLD. Studies have reported that ketogenesis is suppressed in patients with fatty liver disease (7–9). One previous study using isotope tracers reported that acetyl-CoA oxidation through the tricarboxyclic acid (TCA) cycle was upregulated in MAFLD patients, whereas the ketogenic pathway from acetyl-CoA was markedly reduced (7). This suggested that impaired ketogenesis from acetyl-CoA in the liver might be important in the development of MAFLD. However, clinical data on the association between ketogenesis and MAFLD are controversial, as there were also previous studies that showed increased ketone levels in patients with fatty liver disease (10, 11).

In the present study, we aimed to investigate whether impaired ketogenesis is related to MAFLD in patients with type 2 diabetes (T2D). To this end, we recruited newly diagnosed T2D patients and investigated the association between ketogenic capacity based on blood βHB level and MAFLD status using various indices of fatty liver disease.

## Methods

### Study design and population

For this retrospective cross-sectional study, we registered the study subject on the basis of our previous studies (12, 13). Since 2009, we have built a cohort, the diabetes registry of Severance Diabetes Center (a tertiary care hospital in Seoul, Korea) with the patients who underwent a standardized mixed-meal stimulation test on their first visit to our diabetes center. The electronic medical records of patients from April 2017 to March 2022 were reviewed. In the registry protocol, we routinely collected blood samples at 0 and 90 minutes (basal and stimulated, respectively) for glucose, insulin, and C-peptide analyses. Inclusion criteria were patients aged ≥19 years with newly diagnosed T2D based on the 2019 Korean Diabetes Association guidelines (14) and measured serum βHB, which has been available at our center since 2017 from the initial visit. We excluded patients who had undergone organ transplantation or chemotherapy, steroid users, patients who had taken antidiabetic drugs prior to initial blood sampling, and those who visited the emergency room due to hyperglycemia.

A total of 435 patients was ultimately included for analysis in this study. Subjects were classified into two groups based on median initial serum βHB level: those with sufficient ketogenic capacity (intact ketogenesis group) and those without (impaired ketogenesis group).

This study was approved by the independent institutional review board of Severance Hospital (4-2022-1101).

### Measurements and variables

Patient demographics and clinical and biochemical measurements were collected during the study period. The variables gathered were age, sex, body mass index (BMI), use of an antidiabetic drug, hypertension, and blood chemistry.

MAFLD was assessed using previous validated liver steatosis prediction models: hepatic steatosis index (HSI), NAFLD liver fat score (NLFS), Framingham Steatosis Index (FSI), Zhejian University (ZJU) index, and Chinese NAFLD score. The equations are described in **Supplementary Table 1**. HSI and NLFS are well-validated models to detect hepatic steatosis in the general population (15). FSI is a clinical model that includes metabolic parameters such as BMI, diabetes diagnosis, and good discrimination in the National Health and Nutrition Examination Survey III cohort (16). The diagnostic performance of the ZJU index and Chinese NAFLD score was verified in several studies, especially in an Asian cohort including Japanese and Chinese subjects (17, 18).

Serum βHB, the most abundant form of ketone body, was measured before initiating diabetes medication to determine the subject’s ketogenic capacity. Fasting serum βHB concentration was assessed by an enzymatic assay using a commercial reagent from Landox Laboratories Ltd. (County Antrim, UK) and an Atellica CH 930 analyzer (Siemens Healthcare Diagnostics, Marburg, Germany). βHB values measured below the lower limit of the assay were expressed as 0. Blood samples for measuring glucometabolic parameters including serum glucose, insulin, and cholesterol in the fasting state were obtained after overnight fasting. Low-density lipoprotein cholesterol (LDL-C) levels were calculated using Friedewald’s equation in cases of subjects without actual LDL-C measurements, if their blood TG levels were below 400 mg/dL (19). Postprandial insulin level was also measured 90 minutes after the mixed-meal test (Mediwell Diabetic Meal; Meail Dairies Co., Yeongdong-gun, Chungbuk, Korea). The estimated glomerular filtration rate (eGFR) was calculated based on the four-variable Modification of Diet in Renal Disease study equation. Insulin resistance and pancreatic β-cell function were assessed using the homeostasis model assessment of insulin resistance (HOMA-IR) index and HOMA- β.

### Statistical analysis

Glucometabolic parameters and biomarker-based indices to assess hepatic steatosis were compared between the intact ketogenesis and impaired ketogenesis groups. Normally distributed continuous variables were presented as mean (standard deviation), and non-normal continuous variables were presented as median (interquartile range [IQR]). The normality of continuous variables was assessed by the Shapiro-Wilk test. Categorical variables were presented as number with percentage (%). The difference between groups was evaluated using Student’s t-test for continuous variables with normal distribution and Mann-Whitney U-test for continuous variables with non-normal distribution. The frequencies of categorical variables were compared using Pearson’s Chi-square test. The correlation between serum βHB level and each liver steatosis prediction model was assessed by Spearman’s correlation coefficient, which is a statistical method for non-normally distributed variables. In addition, we performed regression analysis to evaluate the clinical significance of initial βHB level for prediction of hepatic steatosis. Logistic regression analysis, a statistical technique used to predict the relationship between independent variables and a binary dependent variable was performed with MAFLD occurrence based on the cut-off values of each MAFLD indices as a dependent variable. The ketogenic capacity used as independent variable in the logistic regression analysis was defined as being divided into intact/impaired based on the median serum βHB level. In the adjusted model, age, sex, BMI, glycated hemoglobin (HbA1c), low-density lipoprotein cholesterol (LDL-C), HOMA-IR, and HOMA-β were adjusted. P-values <0.05 were considered statistically significant. Statistical analyses were performed using R software version 3.6.3 (R Project for Statistical Computing, Vienna, Austria).

## Results

### Clinical and laboratory characteristics of patients

The baseline characteristics of 435 newly diagnosed T2D patients categorized according to level of βHB are shown in **Table 1**. Subjects were divided into intact (n = 226) and impaired (n = 209) ketogenesis group. The median age of the study subjects was 54 years (IQR, 44 to 63) and 62.8% were men. The median serum βHB level was 0.11 (0.0-0.2) mmol/L. Compared to the impaired ketogenesis group, patients with intact ketogenesis were significantly younger and showed non-significantly lower BMI. There were no significant differences in aspartate aminotransferase, alanine aminotransferase, and eGFR between the two groups. Fasting blood glucose level and HbA1c level was significantly higher in the intact group than the impaired group. Moreover, the intact ketogenesis group showed markedly lower fasting and postprandial insulin levels as well as lower HOMA-β and HOMA-IR, suggesting decreased insulin secretory function, better insulin sensitivity, and poor glycemic control. The blood level of TG was lower and LDL-C was higher in the intact ketogenesis group.

**Table 1:** Characteristics of the study population according to baseline βHB.

	Intact ketogenesis	Impaired ketogenesis	*P*-value
	(N=226)	(N=209)	
Demographic			
**Age (years)**	**50.5 [41.0**–**63.0]**	**57.0 [48.0**–**64.0]**	**0.001**
Sex (Male, n (%))	142 (62.8%)	131 (62.7%)	>0.999
HTN	82 (36.3%)	90 (43.1%)	0.178
BMI (kg/m^2^)	25.8 [23.2–28.3]	26.5 [24.2–28.9]	0.146
Biochemistry			
AST (IU/L)	24.5 [19.0-35.0]	27.0 [19.0–36.0]	0.325
ALT (IU/L)	28.5 [19.0-46.0]	30.0 [21.0–44.0]	0.755
Total cholesterol (mg/dL)	196.0 [157.0–234.0]	189.0 [156.5–218.0]	0.174
**TG (mg/dL)**	**135.5 [96.0**–**214.0]**	**150.0 [109.0**–**233.0]**	**0.039**
HDL-C (mg/dL)	43.0 [37.0–53.0]	44.0 [39.0–52.0]	0.435
**LDL-C (mg/dL)**	**112.3 [83.5**–**154.0]**	**105.8 [77.0**–**134.4]**	**0.021**
**TG/LDL-C**	**1.2 [0.8**–**1.9]**	**1.4 [1.0**–**2.2]**	**0.009**
eGFR (ml/min/1.73 m²)	89.8 [77.0-105.5]	90.0 [77.0–107.0]	0.806
uACR (mg/g creatinine)	10.7 [5.9-25.5]	11.4 [5.6–29.7]	0.734
Gluco-metabolic parameters			
**Fasting glucose (mg/dL)**	**143.0 [120.0–220.0]**	**136.0 [120.0–172.0]**	**0.046**
**HbA1c (%)**	**8.9 [7.3-11.0]**	**7.6 [7.0**–**9.2]**	**<0.001**
**Fasting C-peptide (ng/mL)**	**2.4 [1.9-3.0]**	**2.7 [2.2-3.6]**	**<0.001**
**Postprandial C-peptide (ng/mL)**	**5.3 [3.8-6.9]**	**6.4 [4.8-8.4]**	**<0.001**
**Fasting insulin (μIU/mL)**	**9.3 [6.1-13.7]**	**11.7 [7.6**–**18.4]**	**<0.001**
**Postprandial insulin (μIU/mL)**	**40.8 [22.9-62.6]**	**52.4 [32.0**–**89.1]**	**<0.001**
**HOMA-IR**	**3.7 [2.2**–**5.6]**	**4.3 [2.8**–**6.6]**	**0.009**
**HOMA-β**	**36.4 [18.7**–**59.0]**	**49.8 [22.6**–**85.2]**	**<0.001**
**βHB (mmol/L)**	**0.2 [0.1**–**0.4]**	**0.0 [0.0**–**0.0]**	**<0.001**
MAFLD indices			
Hepatic steatosis index	38.3 [34.1-42.1]	38.7 [34.6–42.4]	0.666
Hepatic steatosis index ≥36	147 (65.0%)	141 (67.5%)	0.666
**NAFLD liver fat score**	**0.8 [0.1-1.3]**	**0.9 [0.5**–**1.4]**	**0.045**
NAFLD liver fat score >-0.64	219 (96.9%)	205 (98.1%)	0.631
**Framingham Steatosis Index**	**39.4 [20.4-64.3]**	**47.0 [98.6**–**65.2]**	**0.041**
**Framingham Steatosis Index ≥23**	**165 (73.0%)**	**171 (81.8%)**	**0.038**
Zhejian University index	41.0 [36.1-46.4]	40.1 [37.0–43.8]	0.257
Zhejian University index >38	131 (58.0%)	130 (62.2%)	0.422
Chinese NAFLD score	-0.6 [-1.1–0.0]	-0.4 [-1.0–0.1]	0.264
**Chinese NAFLD score >-0.79**	**133 (58.8%)**	**144 (68.9%)**	**0.038**

BMI, body mass index; HbA1c, glycosylated hemoglobin; TG, triglycerides; HDL-C, high-density lipoprotein cholesterol; LDL-C, low-density lipoprotein cholesterol; HOMA-IR, homeostasis model assessment; HOMA-β, homeostasis model assessment-β; βHB, β-hydroxybutyrate; AST, aspartate aminotransferase; ALT, alanine aminotransferase; HTN, hypertension; eGFR, estimated glomerular filtration rate; uACR, urine albumin-creatinine ratio. The bolded items are statistically significant variables.

**Figure 1:**
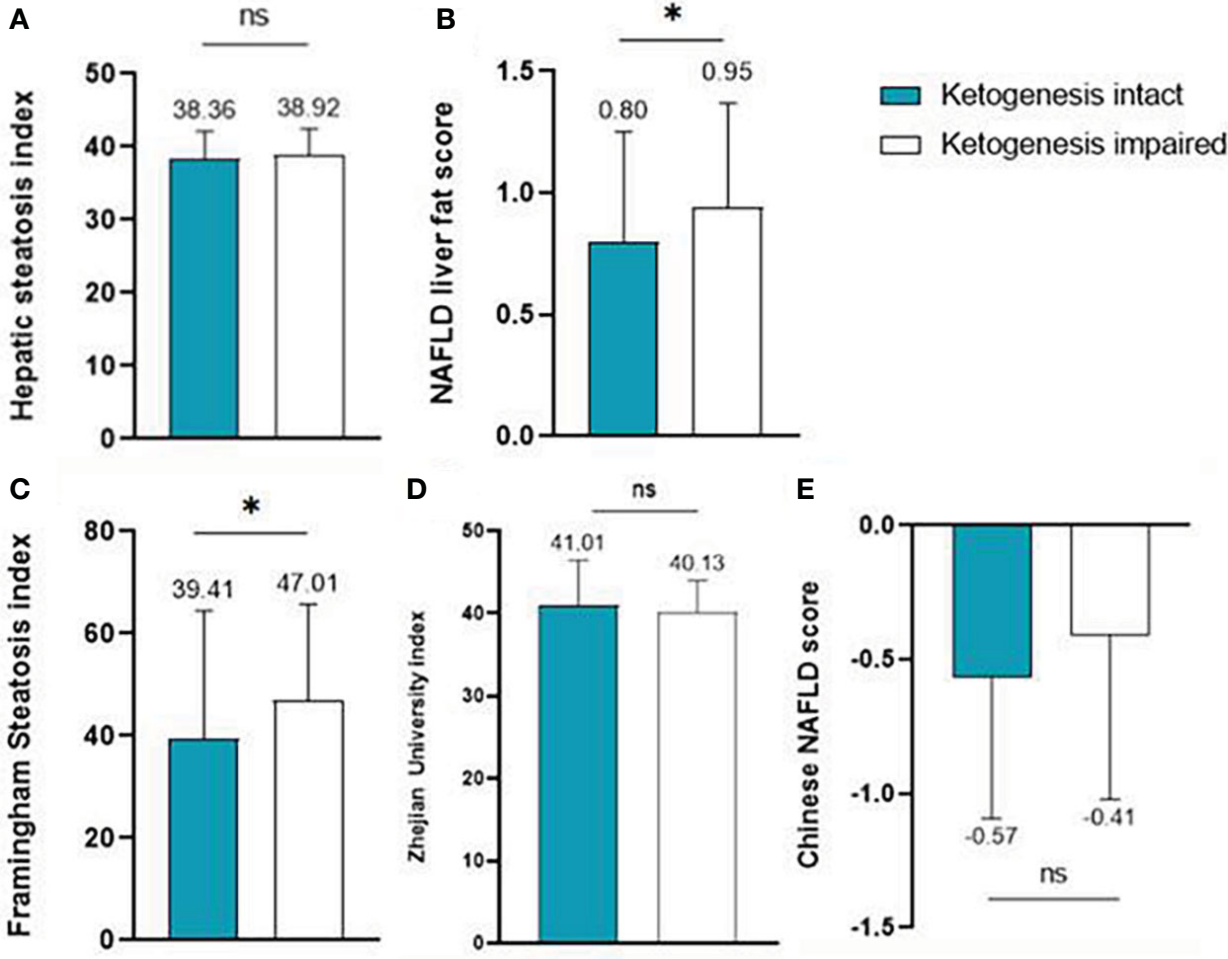
Liver steatosis indices of the intact ketogenesis group and the impaired ketogenesis group: **(A)** Hepatic steatosis index, **(B)** NAFLD liver fat score, **(C)** Framingham Steatosis Index, **(D)** Zhejian University index, and **(E)** Chinese NAFLD score. ns, statistically not significant (P-value ≥ 0.05)*, statistically significant (P-value < 0.05).

The values of the five hepatic steatosis indices of the two groups are described in **Figure 1**. Overall, the intact ketogenesis group showed a tendency to lower hepatic steatosis indices compared to the impaired ketogenesis group. However, significant differences between the groups were found only for NLFS and FSI. In NLFS, the index values of the intact ketogenesis group and impaired ketogenesis group were 0.8 *vs*. 0.9 (p=0.045), and the index value of FSI in groups was 39.4 *vs*. 47.0 (p=0.041). Additionally, the incidence of MAFLD, based on the cut-off values, was lower in the intact ketogenesis group than in the impaired ketogenesis group. Based on assessment by FSI, 165 (73.0%) subjects fulfilled the definition of MAFLD in the intact ketogenesis group whereas 171 (81.8%) subjects in the impaired ketogenesis group (p=0.038). According to the Chinese NAFLD score, 133 (58.8%) in the intact group *vs*. 144 (68.9%) in the impaired group, indicating a significant difference between the two groups (p=0.038).

### Serum βHB level is correlated with lower hepatic steatosis indices

The correlations between serum βHB level and the hepatic steatosis indices were analyzed using Spearman’s correlation coefficient (**Table 2**). There was a significant negative correlation between serum βHB level and hepatic steatosis as assessed by NLFS (r= -0.103, p=0.032) and FSI (r= -0.106, p=0.028). A negative correlation was observed between βHB level and hepatic steatosis based on the other indices, but the relationship was not statistically significant.

**Table 2:** Correlations between βHB and liver steatosis prediction models.

	In all subjects (N=435)	
	β-hydroxybutyrate	
	*r*	p-value
Hepatic steatosis index	**-**0.051	0.281
**NAFLD liver fat score**	**-0.103**	**0.032**
**Framingham Steatosis Index**	**-0.106**	**0.028**
Zhejian University index	-0.068	0.155
Chinese NAFLD score	-0.058	0.226

The bolded items are statistically significant variables.

### Ketogenic capacity is associated with risk of MAFLD incidence

Multivariable logistic regression analysis of the relationship between MAFLD and ketogenic capacity is shown in **Table 3**. To determine the independence of ketogenic capacity as a risk factor for MAFLD, multivariable logistic regression analysis was performed adjusting for covariates of age, sex, BMI, HbA1c, LDL-C, HOMA-IR, and HOMA-β. The analysis showed ketogenic capacity to maintain a significant association with MAFLD as calculated by FSI ([Odds ratio, 0.48; 95% confidence interval 0.25 to 0.91; P=0.025]. With other hepatic steatosis indices, ketogenic capacity tended to be associated with MAFLD incidence, but the relationship was not statistically significant.

**Table 3:** Logistic regression analysis for MAFLD incidence calculated by five liver steatosis models.

In all subjects (N=435)	Hepatic steatosis index		NAFLD liver fat score		Framingham Steatosis Index	
	Adjusted OR	*P*-value	Adjusted OR	*P*-value	Adjusted OR	*P*-value
	(95% CI)		(95% CI)		(95% CI)	
Age (years)	**0.96 (0.94**–**0.99)**	**0.004**	0.99 (0.94–1.04)	0.634	1.02 (0.99–1.04)	0.212
Sex (female *vs*. male)	**2.03 (1.08–3.90)**	**0.030**	1.03 (0.28–4.26)	0.968	0.57 (0.31–1.07)	0.082
BMI (kg/m2)	**2.30 (1.95**–**2.79)**	**<0.001**	1.08 (0.88–1.31)	0.473	**1.93 (1.67**–**2.27)**	**<0.001**
HbA1c (%)	1.00 (0.84–1.19)	0.977	0.89 (0.62–1.31)	0.545	**1.34 (1.12**–**1.62)**	**0.002**
LDL-C (mg/dL)	1.00 (0.99–1.01)	0.508	1.00 (0.99–1.02)	0.695	1.00 (0.99–1.00)	0.595
HOMA-IR	1.02 (0.95–1.10)	0.524	1.10 (0.89–1.58)	0.536	1.00 (0.93–1.09)	0.941
HOMA-β	1.00 (0.99–1.00)	0.160	1.02 (0.99–1.06)	0.225	1.00 (0.99–1.01)	0.925
Ketogenic capacity^*^	1.35 (0.77–2.39)	0.707	0.80 (0.19–2.98)	0.753	**0.48 (0.25**–**0.91)**	**0.025**
	Zhejian University index		Chinese NAFLD score			
	Adjusted OR	*P*-value	Adjusted OR	*P*-value		
	(95% CI)		(95% CI)			
Age (years)	0.98 (0.96–1.01)	0.146	0.99 (0.96–1.02)	0.301		
Sex (female *vs*. male)	1.38 (0.74–2.58)	0.315	0.75 (0.39–1.44)	0.389		
BMI (kg/m2)	**1.81 (1.59**–**2.08)**	**<0.001**	**2.59 (2.15**–**3.21)**	**<0.001**		
HbA1c (%)	**1.56 (1.29**–1.89**)**	**<0.001**	1.03 (0.86–1.22)	0.782		
LDL-C (mg/dL)	1.01 (1.00–1.01)	0.119	1.00 (0.99-1.01)	0.406		
HOMA-IR	**1.43 (1.23**–**1.69)**	**<0.001**	1.04 (0.97-1.13)	0.268		
HOMA-β	**0.98 (0.97–0.99)**	**<0.001**	0.99 (0.99-1.00)	0.101		
**Ketogenic capacity**	0.54 (0.29–1.00)	0.051	0.57 (0.29-1.08)	0.089		

BMI, body mass index; HbA1c, glycosylated hemoglobin; LDL-C, low-density lipoprotein cholesterol; HOMA-IR, homeostasis model assessment; HOMA-β, homeostasis model assessment-β

^*^Ketogenic capacity is defined as being divided into intact/impaired based on the median serum βHB level. The bolded items are statistically significant variables.

## Discussion

In this cross-sectional study, we focused on ketogenic capacity and MAFLD with glucometabolic disorders in patients with T2D. We found that T2D patients with intact ketogenesis showed better hepatic steatosis indices, especially FSI and NLFS. After multivariable logistic regression analysis, ketogenic capacity showed significance in predicting the extent of MAFLD as calculated by FSI. Most of the hepatic steatosis indices other than FSI tended to be correlated with ketogenic capacity but did not show significance in regression analysis. This may be because our study included the large number of newly diagnosed and drug-naïve T2D patients with mild or early stage of MAFLD, as shown in the normal mean liver enzyme levels and slightly increased hepatic steatosis indices. In addition, since the variables used in each index are different, it is believed that differences in sensitivity may have occurred.

In the current study, subjects with intact ketogenesis showed significantly lower insulin resistance without a significant obesity difference compared to those with impaired ketogenesis. The association between insulin resistance and MAFLD has already been well established, and it is thought to have influenced on the pathogenesis of MAFLD in the impaired ketogenesis group. In addition, relative hyperinsulinemia in the impaired ketogenesis group might be due not only to insulin resistance but also to lower hepatic insulin clearance, which has been previously reported to be associated with MAFLD (20). With respect to dyslipidemia in intact ketogenic patients with T2D, higher level of LDL-C and lower TG level were observed in the intact ketogenesis group. The most common pattern of dyslipidemia in T2D is hypertriglyceridemia and reduced HDL cholesterol level. T2D itself does not significantly increase level of LDL-C, but the small dense LDL particles are increased in T2D (21). As a possible explanation for the lipid profile of the intact ketogenesis group, it might be assumed that the pathway in which acetyl-CoA is converted to acetoacetyl-CoA during ketogenesis to result in cholesterol synthesis is more dominant than the pathway in which acetyl-CoA is converted to malonyl-CoA to produce TG in the T2D patients with intact ketogenesis (5, 6).

Ketogenesis or ketone body has been of interest since studies have shown that ketogenesis is associated with better metabolic outcomes. To apply this notion to lifestyle modification, ketogenic, low-carbohydrate diets have been reported to have a significant weight loss effect as well as a glucose-lowering effect (22–24). The ketogenic diet has also demonstrated its beneficial effects on MAFLD (25). In addition, some researchers have reported that subjects with intact ketogenesis, who can produce sufficient ketone bodies during fasting periods, have better metabolic features or clinical outcomes than those who cannot. A cross-sectional study using health check-up data showed that the presence of ketonuria after fasting was associated with metabolic superiority, such as lower body weight, waist circumstance, blood pressure, and blood glucose level (26). In a longitudinal prospective study, healthy individuals with spontaneous fasting ketonuria had a lower risk of incident diabetes (27). However, discrepant reports have been made on the association between ketogenesis and fatty liver disease. A longitudinal study using a cohort of 153,076 nondiabetic Korean subjects reported that fasting ketonuria was associated with reduced risk of incident hepatic steatosis (28). Similarly, a cross-sectional study reported that fasting ketonuria imparted reduced risk for advanced liver fibrosis in MAFLD patients (29). In contrast, Dutch researchers analyzed cohort data and reported that subjects with suspected fatty liver disease had higher blood levels of ketone bodies (11). Several previous studies showed that ketone levels were increased in patients with prediabetes or diabetes (30, 31), which was closely related to fatty liver disease. The result of our study in drug-naïve T2D patients is in line with previous studies that reported lower risk of steatosis or fibrosis in nondiabetic subjects with intact ketogenesis (28, 29).

The pathophysiology or mechanism by which intact ketogenesis in patients with T2D might be protective against MAFLD has not been elucidated, although candidate mechanisms have been proposed. First, in the liver, ketogenesis is an efficient pathway for disposal of FAs from peripheral lipolysis. FAs delivered to the liver are converted to acetyl-CoA through β-oxidation, and ketogenesis is the non-oxidative pathway that converts the acetyl-CoA into energy and disposes of it to the peripheral tissues (5, 6). When the ketogenic pathway is impaired, acetyl-CoA is oxidized to CO2 in the TCA cycle; researchers in the United States reported that the alternative TCA cycle was upregulated *via* fasting,while ketogenesis was impaired in patients with fatty liver disease (7). Unlike the ketogenic pathway, the TCA cycle promotes gluconeogenesis/*de novo* lipogenesis and increases hepatic oxygen consumption. This would cause steatosis and oxidative stress on the liver. As a result, when the ketogenic pathway is impaired, the effective pathway for disposal of FAs in the liver is impaired, and the materials for *de novo* lipogenesis increase along with oxidative stress. Second, the ketogenic process induces hepatic peroxisome proliferation-activated receptor α (PPARα) and fibroblast growth factor 21 (FGF21) action that is important for hepatic lipid metabolism (32–34). The PPARα-FGF21 axis plays a critical role in metabolism across a broad spectrum of organs as well as lipid metabolism in the liver (33, 35). Dysregulation of this axis is associated with pathogenesis of MAFLD (35, 36). Therefore, induction of the PPARα-FGF21 axis in a ketogenic state could decrease steatosis. FGF21 signaling also activates hepatic autophagy (35), in which reduction is a significant process in the pathogenesis of MAFLD (37).

Despite the supportive results of our study, several limitations should be considered. First, our study was a cross-sectional analysis with a relatively small sample size. Therefore, an association between ketogenesis and MAFLD could not be concluded. In addition, characteristics of the intact ketogenesis group, such as higher blood glucose levels and the reduced insulin secretory function, may have acted as prerequisites for the ketogenic ability. From that point of view, ketogenesis may act as a compensatory action in T2D patients with decreased insulin secretion. Due to the limitations of a cross-sectional study, this study cannot suggest the causal relationships and mechanisms clearly. Further studies with larger populations and longitudinal design are needed to collect more evidence on this issue. Second, the study did not use imaging modalities such as ultrasonography, computed tomography, or transient elastography to evaluate the presence or extent of MAFLD. Further studies using these modalities are needed. Third, we did not have data on acetoacetate or acetone because there is no measurable method for them in our center. Therefore, we evaluated ketogenic potency with serum βHB, which is the most abundant form of ketone bodies. In the pathogenesis of MAFLD, not only the metabolic component but also the genetic component are important, and the genetic aspect is mainly expressed in the form of hepatic redox state (38). The βHB/acetoacetate ratio is often used as a marker that reflects hepatic mitochondrial redox state but due to the lack of measurement of acetoacetate, we could not use this marker in our study. If this marker can be included in the analysis in the future, it is considered that the mechanistic explanation of our findings will be more clear. In addition, our study included only T2D patients. Therefore, care must be used when applying the results of this study to a nondiabetic population. However, since previous studies showing the association between ketogenesis and MAFLD were conducted on nondiabetic subjects, this limitation also shows the originality and clinical significance of this study. To the best of our knowledge, this study is the first to report the relationship of ketogenic capacity and hepatic steatogenic status in patients with T2D.

## Conclusions

In T2D patients, intact ketogenesis is associated with better MAFLD indices. Previous findings suggesting that ketogenesis is an efficient pathway for the liver to dispose of FAs, induces the PPARα-FGF21 axis, and reduces oxidative stress are considered to support the results. Large-scale prospective studies using imaging modalities are needed in the future.

## Data availability statement

The raw data supporting the conclusions of this article will be made available by the authors, without undue reservation.

## Ethics statement

The studies involving human participants were reviewed and approved by the Institutional review board of Severance Hospital. Written informed consent for participation was not required for this study in accordance with the national legislation and the institutional requirements.

## Author contributions

SL, JB, and B-WL designed the research. DJ contributed to acquisition of data. SL, JB, and B-WL analyzed the data. SL and JB wrote the article. ML, Y-hL, EK, B-SC, and B-WL contributed to revision of the manuscript. All authors contributed to the article and approved the submitted version.
