# Supplementary material for: Impaired ketogenesis is associated with metabolic-associated fatty liver disease in subjects with type 2 diabetes
Source: Front Endocrinol (Lausanne). 2023 Feb 22;14:1124576. doi: 10.3389/fendo.2023.1124576 (PMC9989459; doi:10.3389/fendo.2023.1124576)
Supplement: Supplementary file 1 [file Table_1.docx]

Supplementary Material

**Impaired ketogenesis is associated with metabolic-associated fatty liver disease in subjects with type 2 diabetes**

**Sejeong Lee^1†^, Jaehyun Bae^2†^, Doo Ri Jo^3^, Minyoung Lee^4^, Yong-ho Lee^4^, Eun Seok Kang^4^, Bong-Soo Cha^4^ and Byung-Wan Lee^4^**

^1^Division of Endocrinology and Metabolism, Department of Internal Medicine, CHA Gangnam Medical Center, CHA University School of Medicine, Seoul, Republic of Korea

^2^Division of Endocrinology and Metabolism, Department of Internal Medicine, Catholic Kwandong University College of Medicine, International St. Mary's Hospital, Incheon, Republic of Korea

^3^Department of diabetes mellitus, Biomedical Research Center, Severance Hospital, Yonsei University College of Medicine, Seoul, Korea.

^4^Division of Endocrinology and Metabolism, Department of Internal Medicine, Yonsei University College of Medicine, Seoul, Republic of Korea

†These authors contributed equally to this work and share first authorship

*** Correspondence:**Byung-Wan Lee, MD, PhD
bwanlee@yuhs.ac

# Supplementary Table

Table S1. Prediction models for MAFLD

| **NAFLD prediction model** | Cutoff  value | Equation |
| --- | --- | --- |
| Hepatic steatosis index (HSI) | >36 | = 8×ALT/AST+BMI (+2, if diabetes; +2, if female) |
| NAFLD liver fat score (NLFS) | >-0.64 | =−2.89+1.18×MetS+0.45×diabetes (yes=2/no=0) +0.15× (fSinsulin, μU/L)+ 0.04×AST−0.94×AST/ALT |
| Framingham steatosis index (FSI) | ≥23 | =1/1+e^-x^ ×100 where x =−7.981+0.011×age (years)−0.146×sex (female=1, male=0) +0.173×BMI+0.007×TG(mg/dL)+0.593×hypertension+0.789×diabetes+1.1×ALT/AST ratio ≥1.33 (yes=1, no=0) |
| Zhejian University (ZJU) index | >38, <32 | =BMI+FPG (mmol/L) +TG (mmol/L)+3×ALT/AST (+2, if female) |
| Chinese NAFLD score | >-0.79 | =−4.632+0.303×MetS+0.157×T2DM (yes =2, no=0) +0.078×fSinsulin (μU/L)+0.168×BMI−0.879×AST/ALT |

ALT, alanine aminotransferase; AST, aspartate aminotransferase; BMI, body mass index; MetS, metabolic syndrome; fSinsulin, fasting serum insulin; TG, triglycerides; FPG, fasting plasma glucose.
